# ADAR1-Mediated RNA Editing and Its Role in Cancer

**DOI:** 10.3389/fcell.2022.956649

**Published:** 2022-07-11

**Authors:** Jizhe Liu, Fei Wang, Yindan Zhang, Jingfeng Liu, Bixing Zhao

**Affiliations:** ^1^ The United Innovation of Mengchao Hepatobiliary Technology Key Laboratory of Fujian Province, Mengchao Hepatobiliary Hospital of Fujian Medical University, Fuzhou, China; ^2^ College of Life Science, Fujian Agriculture and Forestry University, Fuzhou, China; ^3^ Fujian Institute of Research on the Structure of Matter, Chinese Academy of Sciences, Fuzhou, China; ^4^ Mengchao Med-X Center, Fuzhou University, Fuzhou, China; ^5^ Fujian Medical University Cancer Hospital, Fujian Cancer Hospital, Fuzhou, China

**Keywords:** ADAR1, RNA editing, cancer, non-coading RNA, adenosine to inosine

## Abstract

It is well known that the stability of RNA, the interaction between RNA and protein, and the correct translation of protein are significant forces that drive the transition from normal cell to malignant tumor. Adenosine deaminase acting on RNA 1 (ADAR1) is an RNA editing enzyme that catalyzes the deamination of adenosine to inosine (A-to-I), which is one dynamic modification that in a combinatorial manner can give rise to a very diverse transcriptome. ADAR1-mediated RNA editing is essential for survival in mammals and its dysregulation results in aberrant editing of its substrates that may affect the phenotypic changes in cancer. This overediting phenomenon occurs in many cancers, such as liver, lung, breast, and esophageal cancers, and promotes tumor progression in most cases. In addition to its editing role, ADAR1 can also play an editing-independent role, although current research on this mechanism is relatively shallowly explored in tumors. In this review, we summarize the nature of ADAR1, mechanisms of ADAR1 editing-dependent and editing-independent and implications for tumorigenesis and prognosis, and pay special attention to effects of ADAR1 on cancers by regulating non-coding RNA formation and function.

## Introduction

**FIGURE 1:**
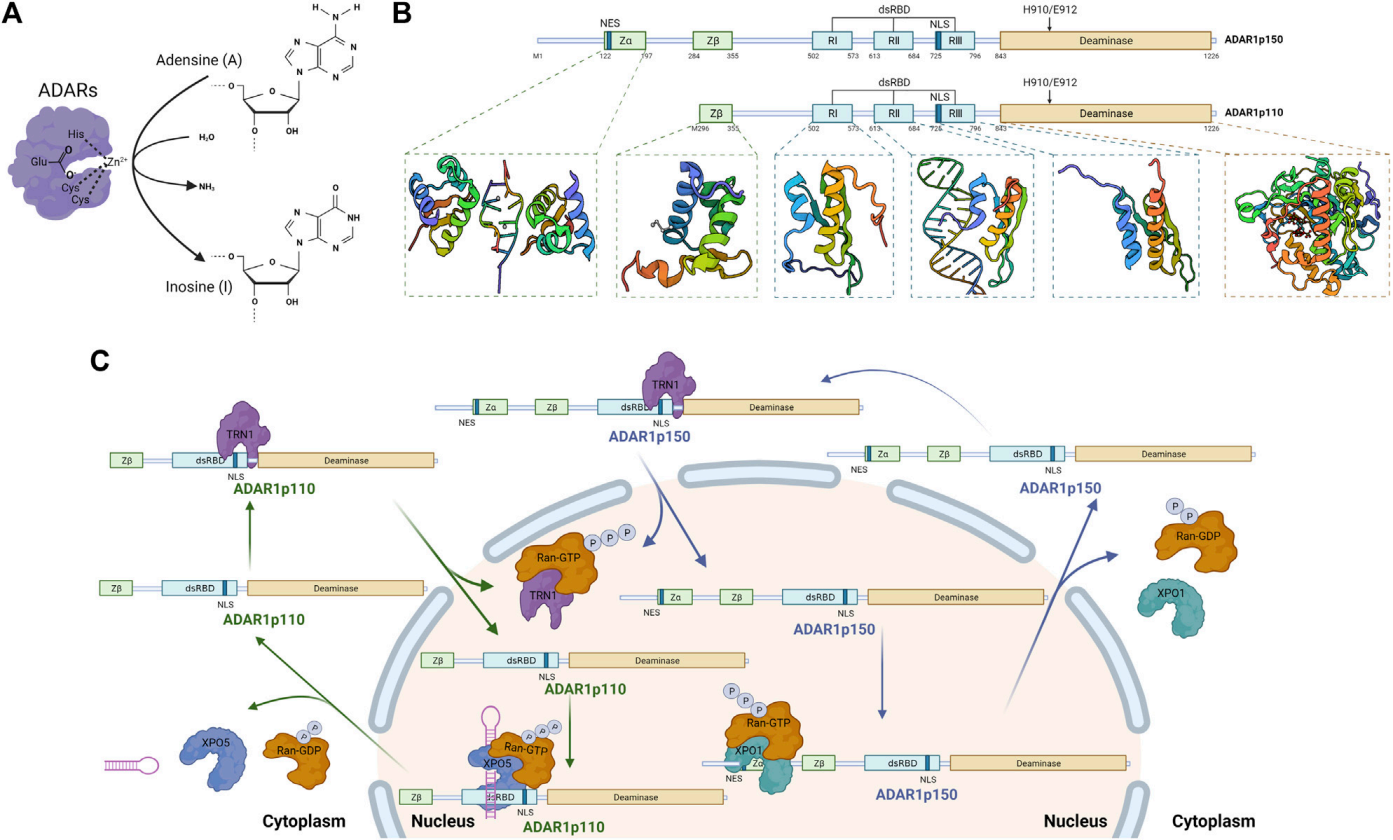
Catalytic function, domain organization, and shuttle mechanism of Adenosine Deaminase Acting on RNA 1 (ADAR1) (Created with BioRender.com). **(A)** RNA editing by ADARs. Alternative RNA editing by C6 deamination of adenosine (A) in the double-stranded RNA region to generate inosine (I), catalyzed by ADARs enzymes. **(B)** Schematic of the domain structure of two isoforms of ADAR1. ADAR1p150 and ADAR1p110 share identical sequence except for an additional N-terminal sequence for ADAR1p150, including an additional Zα domain (PDB: 2GDB) (Placido et al., 2007) and the NES which allows for both cytoplasmic and nuclear localization. Both contain a Zβ domain (PDB: 1XMK) (Athanasiadis et al., 2005) that mainly mediate Z-DNA/RNA binding, three dsRBDs domain (PDB: 2LJH, 2L2K, 2MDR) (Stefl et al., 2010; Barraud et al., 2012; Barraud et al., 2014) that mediate dsRNA binding and homodimerization, and a deaminase domain (PDB: 1ZY7) (Macbeth et al., 2005) that is the catalytic center of ADAR1. **(C)** Cellular localization of ADAR1. XPO1 binds to NES located within the Zα structural domain and regulates nuclear export of ADAR1p150 in the presence of RAN-GTP. Nuclear import of ADAR1p110 is mediated by binding of TRN1 to dsRBD3. NLS located in the third dsRBD, a domain common to both ADAR1 isoforms, binds with TRN1 to assist in the targeting of ADAR1 in the nucleus.

A-to-I editing of RNA, a widespread co/post-transcriptional modification in mammals, is catalyzed by adenosine deaminases acting on RNA (ADARs) and has recently been recognized as an essential mechanism of cancer biology (Han et al., 2015; Baker and Slack, 2022). With the continuous increase of next-generation sequencing data, transcriptomics modifications, so-called RNA mutations, are becoming a significant force in promoting the transformation of normal cells into malignant tumors and providing tumor diversity to avoid immune attacks (Nishikura, 2010; Sagredo et al., 2018; Shevchenko and Morris, 2018). ADARs were first discovered in *Xenopus laevis* oocytes and embyros (Bass, 1987; Quin et al., 2021), confirming mediating editing of adenosine-to-inosine (A-to-I) in double-stranded (ds) RNA in mammals (Xu and Öhman, 2018). This edit acts as a functional A-to-G mutation by hydrolytic deamination at C6 of adenosine, changing it to inosine in the region of dsRNA (Figure 1A) (Nishikura, 2016).

By hydrolytically deaminating adenosine’s 6-position, ADARs catalyze the deamination of adenosine to inosine (Polson et al., 1991). An important target for A-to-I RNA editing is a dsRNA derived from inverted Alu repetitive elements (Alu dsRNA) (Athanasiadis et al., 2004; Bazak et al., 2014). Since cells interpret inosine (I) as guanosine (G), A-to-I editing can lead to non-synonymous codon changes in the transcript and alternative splicing (Walkley and Li, 2017; Eisenberg and Levanon, 2018). In addition, ADARs can bind introns or 3′ untranslated regions (UTRs) to regulate the expression level of relative coding regions (Daniel et al., 2015; Sagredo et al., 2018). Still, it also affects targeting and disrupts the maturation of non-coding consequences, mainly microRNA, lncRNA, and circRNA (Velazquez-Torres et al., 2018; de Santiago et al., 2021; Shen et al., 2021). As a result of ADAR-mediated editing, which arises from mismatched A-to-I signatures after reverse transcription, more than 4 million edited positions have been identified (Bazak et al., 2014; Gallo et al., 2017).

Mammals have three ADAR genes, ADAR1 (also called ADAR, DRADA), ADAR2 (also called ADARB1), and ADAR3 (also called ADARB2), with specific structure, location, and function differences (Xu and Öhman, 2018; Shen et al., 2022). Currently, ADAR1 and ADAR2 have been shown to be expressed throughout most tissues and to be catalytically active, and the former mediates more editing events (Hogg et al., 2011). However, ADAR3 is specifically expressed in the brain and its function is only mentioned in a few articles (Melcher et al., 1996; Chen et al., 2000; Walkley and Li, 2017; Samuel, 2019). In glioblastoma, ADAR3 has been reported to be upregulated to competitively inhibit RNA editing at the Q/R site of *GRIA2* by ADAR2 (Oakes et al., 2017; Wang et al., 2018). And these genes of the ADAR family have similar domain structures, mainly composed of a deaminase domain and two or three dsRNA binding domains (dsRBDs) (Erdmann et al., 2021; Quin et al., 2021). The deaminase domain of both ADAR1 and ADAR2 catalyzes the hydrolytic deamination of adenosine to inosine in dsRNA, while the catalytic activity of ADAR3 has not been reported so far (Chen et al., 2000; Nishikura, 2016; Oakes et al., 2017; Samuel, 2019). The dsRBD (∼65 amino acids) binds directly to dsRNA through its own structural domain. In spite of the fact that both ADAR1 and ADAR2 had been shown to be associated with tumor progression, editing events regulated by ADAR1 are more strongly correlated with cancer development (Maas et al., 2001; Paz-Yaacov et al., 2015). There could be two main reasons for this result. One is that the enrichment of ADAR1 far exceeds that of ADAR2. ADAR1 expression was seen in nearly all tissues, while ADAR2 was most expressed in the brain, but more minor in other tissues (Paz-Yaacov et al., 2015). Significantly, ADAR1 is absolutely required, and ADAR1-null mice die during embryonic life due to extensive apoptosis and defective hematopoiesis (Hartner et al., 2004; Wang et al., 2004). Furthermore, two is that human Adar1 maps to a single locus on chromosome 1q21, whose amplification is the most common cytogenetic abnormalities in multiple types of cancer and a poor prognostic factor (Knuutila et al., 1998; Jiang et al., 2012; Teoh et al., 2018).

ADARs editing is critical for survival in mammals, and its dysregulation may contribute to cancer development (Ramírez-Moya et al., 2021). The function of ADAR1-mediated RNA editing in immunity, especially innate immunity, has been summarized in many reviews (Lamers et al., 2019; Quin et al., 2021; Song et al., 2022), so this article will not address this aspect of the function. In addition to its editing role, ADAR1 can also play an editing-independent role. Regardless of whether the stability of ADARs regulatory RNAs is dependent on RNA editing, RNA binding is essential for their action (Wang I. X et al., 2013). Still, it will focus on the role of ADAR1 in cancer. For example, ADAR1 is more abundant in lung, liver, esophageal and chronic myelogenous leukemia, and with few exceptions, it promotes cancer progression (Jiang et al., 2012; Qin et al., 2014; Zhang et al., 2018; Sun et al., 2020). This review will focus on the structure and regulatory mechanisms of the ADAR1 enzymes and the relationship between aberrant editing of specific substrates and tumorigenesis, especially non-coding RNA.

## Nature of Adenosine Deaminase Acting on RNA 1

The human ADAR1 gene spans approximately 40 kb and includes 17 exons, and the transcript level of ADAR1 is increased by IFN or pathogen stimulation (George et al., 2011). ADAR1 has two protein isoforms, full-length ADAR1p150 (150 kDa) and shorter ADAR1p110 (110 kDa) (Figure 1B) (Sun et al., 2021). Both contain a nuclear localization signal (NLS), allowing nuclear localization (Nishikura, 2016; Baker and Slack, 2022). In addition, ADAR1p150 contains a nuclear export signal (NES), accordingly shuttles between the nucleus and cytoplasm and is predominantly cytoplasmic (Xu and Öhman, 2018; Sun et al., 2021; Baker and Slack, 2022). Both isoforms contain a Zβ domain, three dsRNA-binding domains and a deaminase catalytic domain (George et al., 2011). 1) Zβ domain is a Z-DNA/RNA binding domain which NLS locates in (Athanasiadis et al., 2005); 2) dsRNA-binding domains (RI, RII, and RIII, ∼65 amino acids), which have an α-β-β-β-α configuration, make direct contact with dsRNA (Thomas and Beal, 2017); 3) deaminase domain is the catalytic center of ADAR1, in which the E912A point mutation in ADAR1p150 (E617A for ADAR1p110) disrupts catalytic deaminase activity (Wang I. X et al., 2013; Lamers et al., 2019; Baker and Slack, 2022). Moreover, besides Zβ domain, ADAR1p150 contains Zα domain, another Z-DNA/RNA binding domain, which may have affected its bonding preferences (Nishikura, 2016; Sun et al., 2021). Notably, shorter ADAR1p110 is constitutively expressed in ubiquitous types of cells, whereas full-length ADAR1p150 is expressed only when some activators stimulate cells, such as type I and type II IFN (Rice et al., 2012; Li et al., 2016). Among the inverted retrotransposon repeats of the long spacer nuclear element (LINE) and short spacer nuclear element (SINE) families are a number of endogenous long dsRNAs that are thought to be key-acting substrate RNAs for ADAR1 (Liddicoat et al., 2015). Moreover, the Alu motif mentioned above is a member of the SINE family and is also one of the sites found to be subject to the greatest extent and number of ADAR1 edits (Nishikura, 2010; Liddicoat et al., 2015; Nishikura, 2016).

Although ADAR1p150 is primarily localized in the cytoplasm and ADAR1p110 is predominantly localized in the nucleus, both ADAR1p150 and ADAR1p110 shuttle between the nucleus and cytoplasm (Figure 1C) (Strehblow et al., 2002; Desterro et al., 2003). The nuclear export factor exportin 1 (XPO1, also known as CRM1) binds to the nuclear export signal (NES) located within the Zα structural domain and regulates nuclear export of ADAR1p150 in the presence of RAN-GTP (Poulsen et al., 2001). The binding of transport protein 1 (TRN1) to dsRBD3 mediates nuclear import of ADAR1p110, a process that is inhibited by dsRNA binding (Barraud et al., 2014). The nuclear localization signal (NLS) located in the third dsRBD, a domain common to both ADAR1 isoforms, binds with TRN1 to assist in the targeting of ADAR1 in the nucleus (Fritz et al., 2009).

## RNA Editing-Dependent of Adenosine Deaminase Acting on RNA 1 and Tumorigenesis and Progression Mechanisms

ADAR1 is frequently amplified in many diverse types of cancers with elevated activity (Fritzell et al., 2018), including hepatocellular carcinoma, non-small cell lung cancer, thyroid cancer, pancreatic cancer, esophageal cancer, cervical cancer, and multiple myeloma, and consistent with increased RNA editing levels of its substrates (Chen et al., 2013; Han et al., 2015; Paz-Yaacov et al., 2015; Chen Y et al., 2017; Hu et al., 2017; Sun et al., 2020). Conversely, decreased ADAR1 expression was observed in metastatic melanoma, invasive breast cancer, and kidney cancers (Shoshan et al., 2015; Gumireddy et al., 2016). Most RNA editing sites are located in noncoding regions or noncoding sequences and therefore do not result in changes in protein sequence or expression (Fritzell et al., 2018; Quin et al., 2021). However, a small number of coding events still occur in the coding region, thus affecting gene expression, or even if RNA editing occurs in non-coding sequences, it may indirectly regulates gene expression through mechanisms such as affecting the function of non-coding RNAs. The focus of this section is on this particular editing events by ADAR1 catalyzed, which contribute significantly to cancer development and metastasis. And the content of this section is divided into four main parts according to the differences in catalytic substrates, coding gene, intron, 3′ UTR, and three non-coding RNAs, including microRNA, lncRNA and circRNA. In most cases, increased ADAR1 expression and/or activity promotes cancer generation and progression; while in a few cancers, low expression and/or activity of ADAR1 mediating cancer phenotypes (Shoshan et al., 2015; Gumireddy et al., 2016). We have compiled and summarized the prior literature that experimentally confirms the substrate, editing site, and effects of ADAR1 editing on cancer phenotypes (Table 1).

**TABLE 1:** ADAR1 edits specific substrates involved in cancer progression.

Gene	Substrate type	Editing residues	Cancer	Hallmark	References
ADAR1 promoting cancer					
AZIN1	Coding gene	S/G	LIHC^a^	Growth, colony formation, invasion, migration	Chen et al. (2013)
			ESCC^a^		Qin et al. (2014)
			NSCLC^a^		Hu et al. (2017)
			CRC^a^	Growth, colony formation, invasion, migration, stemness	Shigeyasu et al. (2018), Takeda et al. (2019)
BLCAP	Coding gene	Y/C	LIHC^a^	Proliferation, invasion, migration	Hu et al. (2015)
		Y/C; Q/R; K/R	CESC^a^	Invasion, migration	Chen W et al. (2017)
NEIL1	Coding gene	K/R	MM^a^	Growth, metastasis, colony formation	Jiang et al. (2012)
GLI1	Coding gene	R/G	MM^a^	Growth, colony formation, self-renewal	Shimokawa et al. (2013), Lazzari et al. (2017)
ITGA2	Coding gene	NA^a^	LIHC^a^	Invasion, migration	Yu et al. (2019)
CDK13	Coding gene	Q/R	TC^a^	Proliferation, viability, invasion	Dong et al. (2018), Ramírez-Moya et al. (2021)
FAK	Intron	Intron	LUAD^a^	Growth, metastasis, colony formation	Amin et al. (2017)
ARHGAP26	3′ UTR	3′ UTR	BRCA^a^	Growth, malignant transformation	Wang Q et al. (2013)
DHFR	3′ UTR	3′ UTR	BRCA^a^	Proliferation	Nakano et al. (2017)
miR-200b	MiRNA	MiRNA	TC^a^	Proliferation, invasion, migration	Ramírez-Moya et al. (2020)
miRNA-149-3p	MiRNA	MiRNA	MM^a^	Proliferation, growth	Yujie Ding et al. (2020)
LINC00944	LncRNA	LncRNA	BRCA^a^	Growth, colony formation	de Santiago et al. (2021)
PCA3	LncRNA	Duplex with PRUNE2	PRAD^a^	Growth, adhesion, migration	Bussemakers et al. (1999)
circNEIL3	CircRNA	CircRNA	PDAC^a^	Proliferation, metastasis	Olive et al. (2009), Shen et al. (2021)
circARSP91	CircRNA	CircRNA	LIHC^a^	Growth	You et al. (2015), Shi et al. (2017)
hsa_circ_0004872	CircRNA	CircRNA	GC^a^	Metastasis, colony formation	Ma et al. (2020)
ADAR1 suppressing cancer					
GABRA3	Coding gene	I/M	BRCA^a^	Migration/invasion	Gumireddy et al. (2016)
CCNI	Coding gene	R/G	MEL^a^	Activates of TIL	Zhang et al. (2018)
DFFA	3′ UTR	3′ UTR	BRCA^a^	Invasion	Roberts et al. (2018)
miR-455-5p	MiRNA	MiRNA	MM^a^	Growth, metastasis	Shoshan et al. (2015)
miR-378a-3p	MiRNA	MiRNA	MM^a^	Invasion, migration	Velazquez-Torres et al. (2018)
miR-222	MiRNA	MiRNA	MM^a^	Growth and metastasis, invasion	Galore-Haskel et al. (2015)

a NA, not available; LIHC, liver hepatocellular carcinoma; ESCC, esophageal squamous cell carcinoma; NSCLC, non-small-cell lung cancer; CRC, colorectal carcinoma; CESC, cervical squamous cell carcinoma and endocervical adenocarcinoma; MM, multiple myeloma; TC, thyroid cancer; LUAD, lung adenocarcinoma; BRCA, breast invasive carcinoma; PRAD, prostate adenocarcinoma; PDAC, pancreatic ductal adenocarcinoma; GC, gastric cancer; MEL, melanoma.

### Coding Genes

**FIGURE 2:**
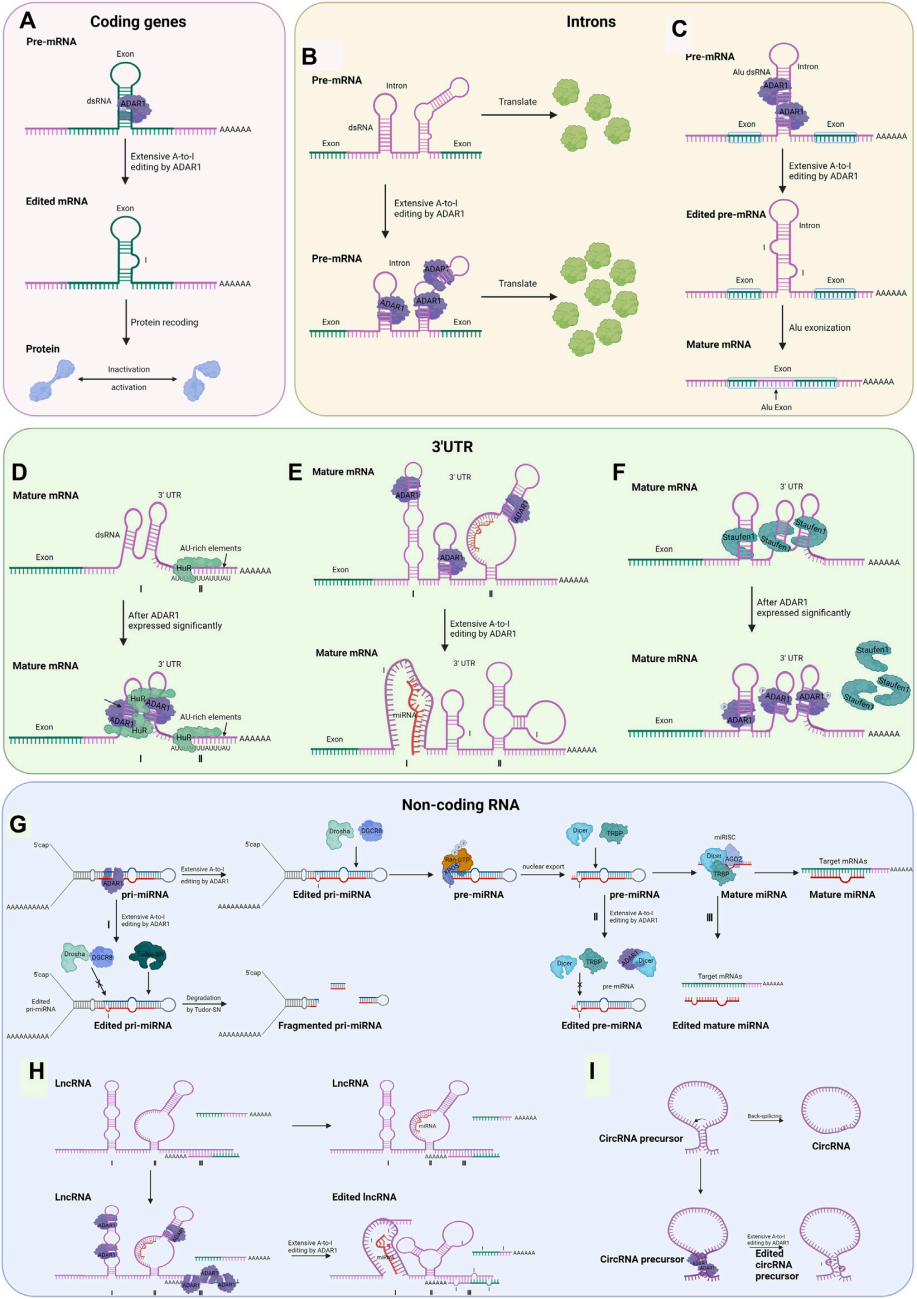
Possible regulatory mechanisms for RNA editing-dependent and editing-independent of ADAR1 involved in tumorigenesis and progression (Created with BioRender.com). **(A)** The dsRNA hairpin structures formed in the exonic region of the encoded gene are recognized by ADAR1. It undergoes A-to-I editing, and splicing machinery interprets inosine as guanosine, which leads to inactivation or activation of the final translation product. **(B)** ADAR1 binds to the intron of pre-mRNA, resulting in increased abundance of exon. **(C)** By editing intronic fold-back dsRNAs with A-to-I editing, splice sites could be created or deleted, leading to the inclusion or exclusion of Alu exons. **(D)** ADAR1 interacts with HuR proteins to coregulate common transcripts. **(E)** Editing of the 3′UTR can change the stability of the mRNA by creating or destroying the binding site of the miRNA. Ⅰ: acquired the ability to bind miRNA; Ⅱ: loss of the ability to bind miRNA. **(F)** ADAR1 competes with Staufen1 protein for the dsRNA binding site, thereby excluding Staufen1 binding and subsequent decaying of antiapoptotic genes in an editing-independent manner. **(G)** The Drosha/DGCR8 complex digests ADAR1-edited pri-miRNAs in the nucleus, generating approximately 70 nt of pre-miRNA intermediates that are translocated to the cytoplasm mediated by exportin-5 (Exp5) and Ran-GTP. The Dicer/TRBP complex undergoes a second cleavage to generate mature miRNAs which target and regulate downstream mRNAs. Because of the editing effect of ADAR1, the process may be aborted at three points. Ⅰ: in pri-mRNA, editing occurs at the RNA site that binds to Drosha and DGCR8, which causes the formation of fragmented pri-miRNA in the action of Tudor-SN; Ⅱ: in pre-miRNA, editing occurs at the RNA site that binds to Dicer and TRBP, or ADAR1 interacts with Dicer; Ⅲ: in mature miRNA, editing occurs at the RNA site that binds to mRNA. The mechanism of miRNA editing by ADAR1 is also applicable to the other two non-coding RNAs and will not be described below. **(H)** Regulatory mechanisms of ADAR1 Editing for dsRNA on lncRNA. Ⅰ: acquired the ability to bind miRNA; Ⅱ: loss of the ability to bind miRNA; Ⅲ: lncRNAs are recognized and edited by ADAR1 after complementary pairing with mRNA bases. **(I)** Editing of Alu repeats can antagonize circRNA formation.

Early mechanistic studies on ADARs focused on causing protein recoding, which potentially modifies the amino acid sequence, thereby leading to decreased activity or acquisition of the encoded protein (Figure 2A). The coding sequence has a double-stranded structure on the exon, which is the region that may be subject to ADAR1-mediated A to I editing. Most A-to-I editing sites are generally found in introns and 3’ UTRs of coding genes, with 1% or fewer occurring in coding exons (Bahn et al., 2012; Wang I. X et al., 2013; Picardi et al., 2015).

Antizyme inhibitor 1 (AZIN1) is the most widely studied ADAR1 substrate in cancer, and edited AZIN1 promotes the development of hepatocellular carcinoma, non-small cell lung cancer, colorectal cancer, esophageal squamous cell carcinoma, and other cancers (Chen et al., 2013; Qin et al., 2014; Hu et al., 2017; Shigeyasu et al., 2018; Xu and Öhman, 2018). For instance, Under the action of deaminase of ADAR1, the serine (S) transforms glycine (G) substitution at residue 367, located in β-strand 15 of AZIN1 in HCC, resulting to its conformational change, inducing a cytoplasmic-to-nuclear translocation, and conferred “gain-of-function” phenotype, such as growth, colony formation, invasion, and migration (Chen et al., 2013). ADAR1 over-edited AZIN1 RNA is an independent risk factor for lymph node and distant metastasis and may serve as a prognostic basis for overall survival and disease-free survival (Shigeyasu et al., 2018). In several of these cancers, AZIN1 has the same editing site and causes similar phenotypic changes in cancer cells (Qin et al., 2014; Hu et al., 2017; Shigeyasu et al., 2018). However, in colorectal cancer, in addition to the above phenotypes, changes in cell stemness are also involved (Shigeyasu et al., 2018; Takeda et al., 2019).

Bladder cancer-associated protein (BLCAP) is a highly conserved gene that plays a tumor-suppressive role in different cancers and is a novel ADAR-mediated editing substrate in hepatocellular carcinoma and cervical cancer (Hu et al., 2015; Chen W et al., 2017). Over-editing of BLCAP was found in 40.1% HCCs compared to adjacent liver tissues. And RNA-edited BLCAP may stably promote cell proliferation by activating AKT/mTOR signal pathway (Hu et al., 2015). In cervical cancers, editing events by ADAR1 alter the genetically coded amino acid in BLCAP YXXQ motif, inducing BLCAP to lose the inhibition to signal transducer and activator of transcription 3 (STAT3) activation, which drives the carcinogenesis progression (Chen W et al., 2017). However, compared with the related normal tissues, BLCAP edited isoforms were reduced in astrocytoma, bladder cancer and colorectal cancer, indicating that the RNA editing level of BLCAP differs in different tumors (Meng et al., 2017).

However, in contrast to the above examples, in some cancers, such as breast cancer, melanoma, RNA editing of ADAR1 inhibits cancer development. In other words, low levels of ADAR1 editing in certain cancers lead to poor prognosis of patients (Gumireddy et al., 2016; Nishikura, 2016; Zhang et al., 2018; Baker and Slack, 2022). ADAR1-edited Gabra3 was only in non-invasive breast cancer and showed that edited Gabra3 reduced the abundance of wild-type Gabra3 on the cell surface and inhibited AKT activation, thereby suppressing breast cancer cell invasion and metastasis (Gumireddy et al., 2016). In melanoma, the ADAR1-edited cell cycle protein I (CCNI)^R75G^ peptide activates tumor-infiltrating lymphocytes (TIL) and promotes TIL killing of cancer cells (Zhang et al., 2018).

To summarize, the above findings suggest that the expression of ADAR1 varies depending on the coding gene and cancer type. Whether ADAR1 overexpression or over-editing promotes or prevents cancer progression depends on the type of editing substrates, their level of expression, and how they participate respectively in regulating malignant changes in cancer cells.

### Intron

The intron in certain coding regions has also been shown to recognize and bind to ADAR1 before being spliced and promote exon expression in an editing-dependent manner. A few articles have reported this approach, and the mechanisms have not been explored in detail (Figure 2B). ADAR1 post-transcriptionally increased the abundance of focal adhesion kinase (FAK) protein by binding and editing to a specific intronic site on chr8: 141,702,274 in FAK transcript, resulting in the increased stabilization of FAK mRNA, thereby promoting mesenchymal properties, migration, and invasion of lung adenocarcinoma (Amin et al., 2017).

RNA editing of Alu-rich intron regions may lead to Alu exonization, which controls mRNA levels and translation efficiency and potentially leads to disease (Figure 2C) (Lev-Maor et al., 2007; Sakurai et al., 2010). However, this modality is only found in G protein-coupled receptor 107 (Athanasiadis et al., 2004), nuclear prelamin A (Lev-Maor et al., 2007) and seryl-tRNA synthetase (Sakurai et al., 2010). However, there is no evidence that ADAR1 mediates cancer-associated gene exonization.

### 3′ Untranslated Region

There are two main ways ADAR1-catalyzed editing on the 3′ UTR influences gene expression regulation. A relatively common one is editing of Alu dsRNA on the 3′ UTR, which binds to and is regulated by microRNA when unedited. The other is dependent on editing to alter the stability of the mature mRNA, mainly achieved by the recruitment of HuR proteins with ADAR1 (Figure 2D) (Song et al., 2016). RNA editing events that occur in the 3′UTR of mRNAs may alter their interactions with miRNAs. Within the 3′ UTRs of mRNAs from nine different types of cancer, Pinto et al. identified over 63,000 editing sites that harbor Alu dsRNAs that serve as recruitment signals for ADAR (Pinto et al., 2018). Several studies have established that RNA editing that occurs in the 3′UTR can create or disrupt miRNA binding sites, thereby altering the mRNA stability of cancer-related genes (Figure 2E) (Liang and Landweber, 2007; Borchert et al., 2009).

For instance, 3′ UTR of Rho GTPase activating protein 26 (ARHGAP26) transcript undergoes ADAR1-catalyzed extensive A-to-I RNA editing (Wang Q et al., 2013). Known as a cancer suppressor, ARHGAP26 activates Rho GTPases (Zhong et al., 2019). Undergone A-to-I editing, the 3′ UTR of ARHGAP26 failed to pair with miR-30b-3p and miR-573, thus translating without interference, thereby promoting breast cancer growth and malignant transformation (Wang Q et al., 2013). As a counterexample, ADAR1, which is much less expressed in breast cancer, targets the 3′ UTR of DNA fragmentation factor subunit alpha (DFFA) transcript, therefore promoting cancer cell invasion (Roberts et al., 2018). DFFA is an inhibitor of caspase-activated DNase (ICAD) that triggers DNA fragmentation during apoptosis (Fawzy et al., 2017). In non-invasive, hormone-responsive breast cancer cell lines, editing of DFFA mRNA by ADFA1 rendered the mRNA unrecognized by miR-140-3p, thereby increasing DFFA levels and inducing apoptosis, whereas lack of editing in highly invasive, triple-negative breast cancer cell lines prevented DFFA from being regulated by miR-1403p (Roberts et al., 2018).

Altered RNA stability is an essential mechanism for ADAR1-mediated regulation of gene expression. ADAR1 recruits and interacts with human antigen R (HuR, gene name *ELAVL1*), a family of RNA-binding proteins selectively binds to single-stranded AU-rich RNA sequences (de Silanes et al., 2003; Meisner et al., 2004), to increase transcript stability (Figure 2D) (Wang I. X et al., 2013). It is estimated that 3′ UTR of up to 8% of all mRNAs contain AU-rich elements, including genes for cancer progression (Shaw and Kamen, 1986; Shaw et al., 2006). Among the 775 genes whose expression levels decreased after ADAR1 knockdown, the genes containing HuR binding sites showed significantly higher expression than the others (Wang I. X et al., 2013), such as MCM4 (Bagley et al., 2012), TMPO(Li et al., 2020), and GSR (McLoughlin et al., 2019).

### Non-Coding RNA

Currently, dysregulated levels of non-coding RNAs (ncRNAs) appear to be reported for every type of cancer, and non-coding transcripts are expected to be the next class of diagnostic and therapeutic tools in oncology (Song et al., 2016; Shen et al., 2022). In addition to RNA editing mediated amino acid substitutions in specific genes contributing to cancer, elevated ADAR1 editing of non-coding RNAs, can also promote cancer progression (Qi et al., 2014; Ramírez-Moya et al., 2020). ADARs can also bind and edit some non-coding genes that contain inverted Alu repeats or LINE of non-coding genes, such as microRNA (miRNA), long non-coding RNA (lncRNA), and circular RNA (circRNA) to suppress non-coding RNA maturation (Torsin et al., 2021).

#### MicroRNA

According to their binding sites along with RNA transcripts, ADAR1 protects mRNA from degradation, regulates miRNA processing, and alters splicing patterns (Xu and Öhman, 2018).

It has been shown that ADAR1 interacts with Dicer or DCGR8 to mediate processing of pre-miRNAs, ultimately leading to microRNA destabilization (Bahn et al., 2015). In this way, the editing of certain microRNA precursors results in lower levels of expression or altered function of mature miRNAs, thereby leading to alterations in certain cancer phenotypes (Figure 2G). In thyroid cancer cells, ADAR1-dependent editing miR-200b exhibits a lower activity against its target ZEB1-3′ UTR, which facilitates the epithelial–mesenchymal transition, thus resulting in proliferation, invasion, and migration of thyroid tumor cells (Ramírez-Moya et al., 2020). In melanoma cells, ADAR1p150 directly interacted with Dicer, an enzyme cleave precursor miRNA (usually 70 nt) to form mature miRNA (around 22 nt), which increased the cleavage rate of pre-miRNA and promoted the loading and maturing of miRNA (Chiosea et al., 2006), thereby promoting the biosynthesis and function of miRNA-149-3p (Yujie Ding et al., 2020). The expression of GSK3α, a direct target of miR-149-3p, is decreased, eventually resulting in proliferation of melanoma cells and inhibited cell apoptosis (Yujie Ding et al., 2020).

Likewise, modification of certain microRNAs by ADAR1 can exert a suppressive effect on cancer, and such miRNAs tend to be less expressed in cancerous tissues with a high metastatic capacity (Shoshan et al., 2015; Velazquez-Torres et al., 2018; Xu and Öhman, 2018). For example, with high levels of A-to-I editing in low metastatic melanoma but not in high metastatic melanoma, edited miR-455-5p lost the binding site of its downstream cancer suppressor protein CPEB1, which resulted in the suppression of melanoma growth and metastasis (Shoshan et al., 2015). Only in non-metastatic melanoma cells, edited miR-378a-3p predominantly binds to and represses the expression of the 3′-UTR of the PARVA oncogene, thereby inhibiting the progression of melanoma to a malignant phenotype (Velazquez-Torres et al., 2018).

#### Long Non-Coding RNA

Recently, LncRNAs have been shown to be central regulators of gene expression in a variety of genes (Guo et al., 2020). The lncRNAs involved in oncogenes and tumor suppressors are modified by A-to-I RNA editing machinery, with modifications that are drastically altered in cancer cells (Silvestris et al., 2020). ADAR1 can change RNA expression levels by interacting with other RNA-binding proteins, such as Dicer (Deng et al., 2019) and HuR (Stellos et al., 2016), the same mechanism as the 3′ UTR and the miRNA described previously. In addition, editing of dsRNA on lncRNA by ADAR1 changes its structure, which affects the binding of downstream target miRNAs (Figure 2H). Exceptional cases are reported in the literature where lncRNAs are recognized and edited by ADAR1 after complementary pairing with mRNA bases (Figure 2H) (Bussemakers et al., 1999).

The expression of ADAR1-regulated lncRNA LINC00944 is immune-related in breast cancer cells. LINC00944 expression was positively correlated to tumor infiltrating T lymphocytes, the age at diagnosis, tumor size, and poor prognosis (de Santiago et al., 2021). In spite of the variations in LINC00944 expression corresponding to up and down regulation of ADAR1, it is unknown the regulatory mechanism of ADAR1 for this lncRNA (de Santiago et al., 2021).

Prostate cancer antigen 3 (PCA3), a long non-coding RNA, is upregulated in human prostate cancer (Bussemakers et al., 1999). PCA3 regulates the level of PRUNE2 through a unique regulatory mechanism that involves the formation of PRUNE2/ADAR1-edited PCA3 double-stranded RNA. Editing by ADAR1 at multiple sites in the PCA3/PRUNE2 duplex results in a reduction of PRUNE2, and an increase in PCA3 expression, therefore increasing in cancer cell the ability of cancer cell, such as proliferation, adhesion and migration (Bussemakers et al., 1999).

#### Circular RNA

As mentioned above, ADAR1 interacts with by binding to the inverse complementary dsRNA, such as Alu repeat region; circRNAs can also interact with it in this way (Chen and Yang, 2015). ADAR1 creates or disrupts splice sites by acting on dsRNA editing, thereby inhibiting circRNA cyclization (Figure 2I) (Zhang and Carmichael, 2001; Fritz et al., 2009; Shevchenko and Morris, 2018).

CircNEIL3 and ADAR1 are upregulated in pancreatic ductal adenocarcinoma (PDAC) cells and tissues, and circNEIL3 as a miRNA sponge leads to down-regulation of miR-432-5p, thereby suppressing the down-regulation effect caused by the 3′UTR interaction of this microRNA with ADAR1 (Xu et al., 2020; Shen et al., 2021). Subsequently, ADAR1 enhances GLI1 editing, which promotes the transcription of target genes, including cyclin D1, cyclin E and Snail, and the cyclin-dependent kinases CDK2, CDK4 and CDK6 afterward decreased (Olive et al., 2009; Wang et al., 2020; Shen et al., 2021). In summary, the circNEIL3/miR-432-5p/ADAR1/GLI1/cyclin D1/CDK axis regulates the proliferation and metastasis of PDAC *via* the downstream GLI1/cyclin D1 and EMT pathway (Olive et al., 2009; Shen et al., 2021).

In addition, ADAR1 has been described to repress circRNA production intensely, and its A-I editing process usually occurs near reverse complementary matches (RCMs) in circRNA flanking introns, A structure essential for circRNA cyclization (Shi et al., 2017; Ma et al., 2020). Through this editing, secondary structures between RCMs could be stronger or weaker respectively depending on whether correcting A:C was mismatched to I(G)-C pairs or I(G) U pairs (Shen et al., 2022).

Androgen receptor (AR), a transcriptional activator of ADAR1 promoter, could suppress circARSP91 expression by upregulating ADAR1 p110, eventually leading to HCC tumor growth both *in vitro* and *in vivo* (You et al., 2015; Shi et al., 2017). Circular RNA hsa_circ_0004872, dramatically downregulated in gastric cancer, molecular sponges miR-224 to upregulate the expression of the miR-224 downstream targets p21 and Smad4 by targeting the 3′-UTR (Ma et al., 2020). Smad4, as a transcription factor, could inhibit ADAR1 expression level by directly binding to the promoter region of ADAR1, thereby further upregulating hsa_circ_0004872 levels. In other words, Smad4/ADAR1/hsa_circ_0004872/miR-224/Smad4 axis regulated tumor size and local lymph node metastasis in gastric cancer (Hansen et al., 2013; Zhu et al., 2017; Ma et al., 2020).

ADAR1 can edit reverse complementary matches (RCM) of ADAR1-regulated circRNAs, altering the secondary structure formed between RCMs within the flanking introns and enhancing the binding of RNA-binding proteins (RBPs) to the site of action (Kristensen et al., 2019; Shen et al., 2022). And the different expression of ADAR1 leads to editing dysregulation of A-to-I RNA editome in multiple cancers, such as HCC, ESCC, CRC, and GC (Qin et al., 2014; Han et al., 2020; Song et al., 2021). These ADAR1-regulated circRNAs are not byproducts of reverse splicing but important molecules that influence cancer development and progression.

## RNA Editing-Independent of Adenosine Deaminase Acting on RNA 1 and Tumorigenesis and Progression Mechanisms

The most direct role of ADAR1 relies on A-to-I editing, which can alter coding sequences, binding motifs, RNA structure, etc., to regulate substrate abundance. However, ADARs have also been shown to have an editing-independent role, as they can also function as RNA-binding proteins independent of catalytic activity (Nishikura, 2016).

In melanoma, ADAR1 controls the expression of integrin beta 3 (ITGB3), a cell surface protein associated with tumor invasion (Pinon and Wehrle-Haller, 2011; Orgaz and Sanz-Moreno, 2013), *via* miR-22 and PAX6 transcription factor at the post-transcriptional and transcriptional levels. The expression and functional output of both FOXD1, which controls miR-22 expression, and PAX6 are independent of RNA editing, therefore promoting growth and invasion of melanoma (Nemlich et al., 2018).

In cytoplasm, binding of the targeted 3′ UTR allows phosphorylated ADAR1p110 to preclude binding of Staufen1 and subsequent decay of the anti-apoptotic gene, thereby promoting survival of stressed cells in an editorially independent manner (Figure 2F) (Sakurai et al., 2017). It has been reported in the literature that Staufen1 has positive or negative effects on disease progression: in some malignancies, upregulated Staufen1 may act as an oncogene and promote cancer progression (Boulay et al., 2014; Xu et al., 2015; Crawford Parks et al., 2017); while in other cancers, Staufen1 may act as a tumor suppressor and inhibit disease progression (Gong and Maquat, 2011; Boulay et al., 2014; Su et al., 2020). Nevertheless, reports on the mechanism of ADAR1 for Staufen1 have not yet appeared in cancer. Interaction of lncRNA with Staufen1 has also been reported in a variety of cancers (Sakurai et al., 2017); however again no mechanism of action of ADAR1 for Staufen1 in tumors has been reported. In HCC, ADAR1 was detected to interact directly with Dicer without editing, resulting in the processing of pre-miR-27a (Qi et al., 2017). Mature miR-27a binds to the 3′-UTR of methyltransferase 7A (METTL7A), a known tumor suppressor, decreasing its expression level (Qi et al., 2017). In addition, ADAR1 can regulate miRNA processing in an RNA-binding but editing-independent manner. ADAR1 can indirectly affect miRNA biogenesis by regulating Dicer expression at the translational level through the Letal-7 gene (let-7) (Zipeto et al., 2016).

## Concluding Remarks and Future Perspectives

In conclusion, we show the structure and regulatory mechanisms of the ADAR1, and the significant role of ADAR1 in regulating many aspects of RNA function in cancers, including regulating the biogenesis of coding gene, intron, 3′ UTR, and three prevalent non-coding RNA, such as miRNA, lncRNA, circRNA. However, many important questions remain in the field including: 1) What are the factors that influence altered ADAR1 expression in cancer, and how do they play a role? 2) There are many editing-independent mechanisms of action in cancer that exist only in theory and for which there is no clear experimental evidence. 3) Up-regulation of ADAR1 expression promotes cancer development and progression in most cases, and down-regulation of ADAR1 in some cases can also achieve cancer progression. How do cancer cells control the abundance of ADAR1 so precisely to enhance their progression? Future investigation of these issues may lead to further discoveries in A-to-I RNA editing.
